# Randomized trial evaluating serial protein C levels in severe sepsis patients treated with variable doses of drotrecogin alfa (activated)

**DOI:** 10.1186/cc9382

**Published:** 2010-12-21

**Authors:** Andrew F Shorr, Jonathan M Janes, Antonio Artigas, Jyrki Tenhunen, Duncan LA Wyncoll, Emmanuelle Mercier, Bruno Francois, Jean-Louis Vincent, Burkhard Vangerow, Darell Heiselman, Amy G Leishman, Yajun E Zhu, Konrad Reinhart

**Affiliations:** 1 Washington Hospital Center, 110 Irving Street NW, Washington DC 20010, USA; 2 Lilly Research Laboratories, Eli Lilly and Company, Lilly Corporate Centre, 893 South Delaware Street, Indianapolis, Indiana 46285, USA; 3 Critical Care Center, Sabadell Hospital, CIBER Enfermedades Respiratorias, Autonomous University of Barcelona, Parc Taulí 1, 08208 Sabadell, Spain; 4 Critical Care Medicine Research Group in Department of Intensive Care Medicine, Tampere University Hospital, Teiskontie 35, Tampere, 33521, Finland; 5 Adult Intensive Care Unit, Guy's and St Thomas' NHS Foundation Trust, Lambeth Palace Road, London, SE1 7EH, UK; 6 Service de Réanimation Polyvalente - CRICS group, Hopital Bretonneau-CHRU, 2 Boulevard Tonnellé, Tours, 37044, France; 7 Service de Réanimation Polyvalente - CIC-P 0801 Inserm - CRICS group, CHRU Dupuytren, Limoges, 87042, France; 8 Department of Intensive Care, Erasme University Hospital, Université Libre de Bruxelles, Route de Lennik 808, 1070, Brussels, Belgium; 9 Department of Anesthesiology and Intensive Care, Friedrich-Schiller University, Erlanger Allee 101, Jena, 07743, Germany

## Abstract

**Introduction:**

Serial alterations in protein C levels appear to correlate with disease severity in patients with severe sepsis, and it may be possible to tailor severe sepsis therapy with the use of this biomarker. The purpose of this study was to evaluate the dose and duration of drotrecogin alfa (activated) treatment using serial measurements of protein C compared to standard therapy in patients with severe sepsis.

**Methods:**

This was a phase 2 multicenter, randomized, double-blind, controlled study. Adult patients with two or more sepsis-induced organ dysfunctions were enrolled. Protein C deficient patients were randomized to standard therapy (24 μg/kg/hr infusion for 96 hours) or alternative therapy (higher dose and/or variable duration; 24/30/36 μg/kg/hr for 48 to 168 hours). The primary outcome was a change in protein C level in the alternative therapy group, between study Day 1 and Day 7, compared to standard therapy.

**Results:**

Of 557 patients enrolled, 433 patients received randomized therapy; 206 alternative, and 227 standard. Baseline characteristics of the groups were largely similar. The difference in absolute change in protein C from Day 1 to Day 7 between the two therapy groups was 7% (*P *= 0.011). Higher doses and longer infusions were associated with a more pronounced increase in protein C level, with no serious bleeding events. The same doses and longer infusions were associated with a larger increase in protein C level; higher rates of serious bleeding when groups received the same treatment; but no clear increased risk of bleeding during the longer infusion. This group also experienced a higher mortality rate; however, there was no clear link to infusion duration.

**Conclusions:**

The study met its primary objective of increased protein C levels in patients receiving alternative therapy demonstrating that variable doses and/or duration of drotrecogin alfa (activated) can improve protein C levels, and also provides valuable information for incorporation into potential future studies.

**Trial registration:**

ClinicalTrials.gov identifier: NCT00386425.

## Introduction

Severe sepsis and septic shock remain associated with substantial morbidity and mortality [1]. Among patients with severe sepsis, protein C levels are often low at the time of diagnosis [2-5]. Temporal changes in protein C levels also appear to parallel the course of disease progression and resolution [6-9]. For example, in patients surviving their episode of sepsis, protein C levels fall and then begin to recover, while in those who eventually succumb, protein C values decline and often remain low [6,10]. Serial alterations in protein C also appear to correlate with disease severity as measured by the development of organ failure and the evolution of those organ failures [11,12].

In PROWESS, a large randomized controlled trial of drotrecogin alfa (activated) (DAA) [3], protein C levels 96 hours after enrollment correlated strongly with eventual outcomes [9]. In patients treated with DAA, protein C levels rose more rapidly and were higher at 96 hours than in subjects randomized to placebo. Nonetheless, in some individuals treated with DAA protein C levels remained low despite DAA therapy or rose initially then fell with the discontinuation of DAA therapy [9,10]. The nexus between protein C measurements, DAA infusion, and eventual outcomes suggests that the current strategy for administering DAA might be improved by titration of therapy based on a patient's individual protein C levels. Presently, the decision to initiate DAA is made based on clinical grounds irrespective of baseline and subsequent protein C levels, and patients are given a fixed dose and duration of DAA (24 μg/kg/hr for 96 hours). Initial protein C levels could also serve as a biomarker to indicate which patients might benefit from DAA [10,13,14,9]. Moreover, the extent and variability in protein C levels in severe sepsis, along with the strong link between the end of DAA administration protein C values and outcomes, suggests that an alternate approach may be warranted [14,15]. Some patients might benefit from either an extended duration of treatment and/or a higher dose of DAA titrated to their unique response and disease evolution, leading to a more individualized, patient-centered paradigm. Such an approach would assume that giving more DAA would result in improved protein C levels, and this in turn would be associated with improved patient outcome.

In order to test the first part of this hypothesis, that a variable dose and/or duration of DAA infusion could alter protein C values, we conducted an exploratory phase 2, double-blind, randomized trial in which patients received either standard DAA therapy or had their DAA dose and/or infusion length altered based on serial protein C levels and the eventual normalization in protein C. We also sought to evaluate the safety of alternate strategies for DAA administration, and to provide additional information critical for the design of possible future studies.

## Materials and methods

### Study patients

From November 2006 to August 2009, we enrolled eligible adult patients (≥18 years old) in this multicenter, randomized, double-blind, parallel, controlled, dose comparison phase 2 study. The study was approved by the ethics committee at each participating center and written informed consent was obtained from all participants or their authorized representatives. The study was compliant with the Declaration of Helsinki and consistent with good clinical practices.

### Selection criteria

Patients were eligible for the study if diagnosed with severe sepsis (presence of a suspected or proven infection) and two or more sepsis-associated organ dysfunctions (cardiovascular, respiratory, renal, hematologic, or metabolic acidosis). Disease diagnostic definitions are provided online in Table S1 in Additional file 1. Exclusion criteria were similar to those used in PROWESS [3] and are detailed in Table S2 in Additional file 1. Main exclusion criteria included documented multiple organ dysfunction >24 hours prior to start of the study drug; body weight <30 kg or >135 kg; platelet count <30,000/mm^3^; active internal bleeding or an increased risk of bleeding. We excluded patients not expected to survive 28 days given a pre-existing uncorrectable medical condition.

### Study design and treatment assignments

A description of the RESPOND study design has previously been published [14] and a simplified study design is depicted in Figure S1 in Additional file 1. Patients diagnosed with at least two organ failures within 24 hours of the start of DAA therapy and protein C deficiency (protein C levels less than the lower limit of normal) were randomized to standard DAA therapy (24 μg/kg/hr infusion for 96 hours) or alternative DAA therapy (higher dose and/or variable duration). Both patient groups received the same common lead-in therapy of 24 μg/kg/hr DAA for the first 24 hours before then receiving their assigned randomized therapy. Based on the 24-hour (Day 1) protein C measurement, determined locally at study hospitals, patients stratified in the moderate deficiency group (protein C levels >1/2 the lower limit of normal) and assigned to alternative therapy, received a standard dose DAA (24 μg/kg/hr) and variable duration infusion for 48 to 168 hours in total. Patients stratified in the severe deficiency group (protein C levels ≤1/2 the lower limit of normal) and assigned to alternative therapy, received a higher dose DAA (30 or 36 μg/kg/hr infusion) and variable duration of infusion for a maximum of 168 hours. Treatment in the alternative arm continued until two consecutive protein C levels (12 hours apart) were greater than or equal to the lower limit of normal ("normalized"). Definitions used to define protein C deficiency are shown in Table S3 in Additional file 1. In the pre-amended protocol (see mortality and safety section of results), if protein C measurements normalized before the completion of the indicated 96 hours of infusion, alternative therapy patients could be switched to a placebo infusion (sterile 0.9% sodium chloride), subject to investigators agreement based on their assessment of clinical improvement. Patients randomized to standard therapy, stratified either in the moderate or severe deficiency groups, all received a standard dose and duration of DAA (24 μg/kg/hr infusion for 96 hours). Patients who entered the study without decreased protein C levels (protein C levels greater than the lower limit of normal) at 24 hours from two organ failure evolution, were followed in a nondrug-interventional arm (results not included in this manuscript), and received normal care (which may have included DAA) at the discretion of the investigator.

DAA (Xigris^®^, Eli Lilly and Co., Indianapolis, IN, USA) was supplied as a sterile freeze-dried product in glass vials and administered by site personnel as a continuous intravenous infusion.

An interactive voice response system (IVRS) provided patient randomization, performed as block randomization stratified by investigator site. Patient's treatment assignments and dosing levels were prepared by an unblinded pharmacist or designee through the IVRS. Patients, investigators, and sponsor (Eli Lilly and Company) were blinded throughout the study unless involved in safety monitoring or data monitoring committee (DMC) activities. The study drug delivery system was shrouded to enhance blinding. A locally obtained placebo infusion of sterile 0.9% sodium chloride was used as necessary to ensure study drug infusion durations were indistinguishable between treatment groups.

### Objectives and study measurements

The primary objective was to test the hypothesis that alternative therapy would result in a greater increase in protein C level from study Day 1 to study Day 7 compared with standard therapy with DAA. Secondary objectives included: safety profile of higher doses and longer infusions of DAA assessed by adverse events and bleeding; change in protein C level by subgroup (moderate and severe protein C deficiency patients); and 28-day all-cause mortality. Base-line demographics and clinical characteristics were also collected.

While patients in the intervention arm had their DAA treatment adjusted based on local protein C measurements, protein C levels for analysis of the primary efficacy measure were measured at a central laboratory (Covance, Indianapolis, IN, USA) using a Stago clotting (Staclot) protein C activity-based test (Diagnostica Stago, Asnières-sur-Seine, France). These central laboratory results were not available to investigators and not used for treatment stratification. Protein C levels determined locally, used to stratify patients as moderate or severe and make decisions related to completion of study drug infusion, were measured by a Stago chromogenic (Stachrom) protein C activity-based test, or by a point-of-care antibody-based protein C test developed by Biosite Incorporated (San Diego, CA, USA) specifically for this study. These assays are not significantly interfered with by the administration of DAA.

All patients were followed for at least 28 days from the start of the infusion or until hospital discharge, death, or 90 days, if the patient remained in the study hospital at study Day 28.

### Statistical analysis

Based on data from PROWESS [3], it was estimated that 422 patients treated with randomized therapy, would provide 80% power to detect a mean difference in protein C change of 7.5% (absolute activity) between study Day 1 and study Day 7 between treatment groups. Planned interim analyses by an internal DMC were included as a safety evaluation to be conducted before the dose of DAA was increased from 30 to 36 μg/kg/hr in the alternative arm in patients with severe protein C deficiency. Data analyses were carried out according to a prospectively defined analysis plan, and all treatment effect tests were conducted at a two-sided alpha level of 0.05. The predefined primary analysis population were patients who received any amount of randomized therapy (primary efficacy population) with combined alternative therapy and standard therapy arms. The mean change in protein C from study days 1 to 7 in the two treatment groups was compared using an unadjusted 2-sample t-test and missing data imputed using the last observation carried forward method. Hospital and 28-day mortality rates in each treatment group were compared using Fisher's exact test. The proportion of patients who experienced adverse events was compared between treatment groups using Fisher's exact test.

## Results

### Patients

**Figure 1:**
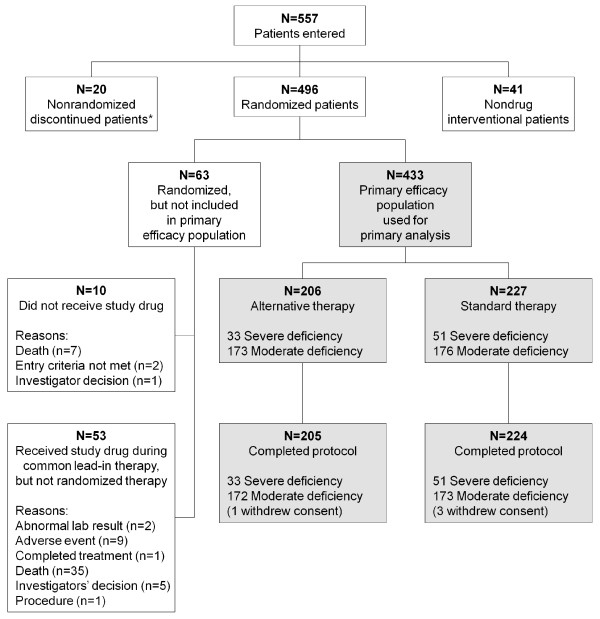
**Patient disposition and study flow diagram of patients**. *Patients who signed informed consent, but did not proceed to randomization or the nondrug-interventional arm.

A total of 557 patients were entered into the study from November 2006 to June 2009, conducted at 52 hospitals in 11 countries. Of these, 496 patients were randomly assigned to treatment; 433 received any amount of randomized therapy (received after 24 hour common lead-in therapy) and defined the primary efficacy population used for efficacy analyses (Figure 1). A number of assumptions in planning this study were not realized (Table S4 in Additional file 1). Namely, a greater than expected number of patients were stratified as moderately protein C deficient (80% actual *vs *60% expected) and thus fewer patients than expected were stratified as severely protein C deficient (20% actual *vs *40% expected). In the severe deficiency strata, it was planned to test four higher doses (30, 36, 42, and 48 μg/kg/hr) in the alternative therapy arm. However, because of the smaller than expected number of patients in the severe deficiency strata, only two doses could be tested (30 and 36 μg/kg/hr). This in combination with a smaller than expected number of alternative therapy patients requiring ≥97 hours to normalize their protein C level, led to a large proportion of patients in the alternative therapy group receiving, in effect, standard therapy. As a result, not as many patients as anticipated received longer infusions (46% actual *vs *70% to 75% expected), or higher doses of DAA. These results are also reflected in the exposure data. The largest difference in drug exposure (more than double) was seen in patients in the severe protein C deficiency strata, where alternative therapy patients had a mean exposure of 4,196.2 μg/kg and a mean infusion duration of 126.5 hours, compared to 1,991.5 μg/kg and 77.1 hours, respectively, for standard therapy patients. In the moderate protein C deficiency strata, the difference was less marked; alternative therapy patients had a mean exposure of 2,700.6 μg/kg and a mean infusion duration of 100.5 hours compared with 2,336.5 μg/kg and 90.0 hours, respectively, for standard therapy patients. In the moderate protein C deficiency strata the median infusion duration was 96 hours in both treatment groups; about half of the alternative therapy patients had an infusion duration of 96 hours or less. The longest median infusion duration was in the alternative therapy group in the severe protein C deficiency strata (128 hours).

Baseline characteristics, and sites and causes of infection at baseline (Table 1 and 2) were largely similar between the standard and alternative therapy groups. A history of thrombosis was the only statistically significant difference between the treatment groups (*P *= 0.009). There were some statistically nonsignificant but noteworthy imbalances: the alternative therapy group had a greater percentage of patients requiring vasopressor support and a greater percentage of patients classed with severe protein C deficiency, with the lung as the primary site of infection, and the standard therapy group had a greater percentage of patients with renal dysfunction, with the abdomen as the primary site of infection, that were receiving insulin therapy, had a history of hypertension and a history of diabetes.

**Table 1:** Summary of baseline characteristics of the primary efficacy population

Variable	Alternative therapy (*n *= 206)	Standard therapy (*n *= 227)	Total (*n *= 433)	*P*-value*
Age, mean ± SD	61.9 ± 14.4	62.3 ± 16.1	62.1 ± 15.3	0.480
Male, *n *(%)	130 (63.1)	137 (60.4)	267 (61.7)	0.556
Caucasian, *n *(%)	189 (91.7)	204 (89.9)	393 (90.8)	0.172
European, *n* (%)	144 (69.9)	159 (70.0)	303 (70.0)	0.974
Recent surgery, *n *(%)	61 (29.6)	68 (30.0)	129 (29.8)	0.575
Number of organ dysfunctions, *n *(%):				0.759
2	55 (26.7)	62 (27.3)	117 (27.0)	
3	88 (42.7)	99 (43.6)	187 (43.2)	
4	54 (26.2)	52 (22.9)	106 (24.5)	
5	9 (4.4)	14 (6.2)	23 (5.3)	
Number of organ dysfunctions, mean ± SD	3.08 ± 0.84	3.08 ± 0.86	3.08 ± 0.85	0.97
Organ dysfunction criteria, *n *(%):				
Cardiovascular	199 (96.6)	220 (96.9)	419 (96.8)	0.853
Respiratory	175 (85.0)	185 (81.5)	360 (83.1)	0.338
Renal	114 (55.3)	139 (61.2)	253 (58.4)	0.214
Hematology	37 (18.0)	36 (15.9)	73 (16.9)	0.560
Metabolic	110 (53.4)	119 (52.4)	229 (52.9)	0.839
Time of onset of 2^nd ^OD to start of drug infusion, hr ± SD	15.0 ± 7.0	15.3 ± 7.0	15.2 ± 7.0	0.810
Total SOFA, mean ± SD	8.65 ± 2.70	8.38 ± 2.83	8.51 ± 2.77	0.657
APACHE II score, mean ± SD	26.15 ± 7.31	26.34 ± 7.70	26.25 ± 7.51	0.854
DIC, average mean score ± SD	3.95 ± 1.14	4.01 ± 1.16	3.98 ± 1.15	0.62
Use of vasopressor, *n *(%)	183 (88.8)	190 (83.7)	373 (86.1)	0.122
D-dimer level (mg/L), mean ± SD	7.31 ± 8.47	8.29 ± 9.48	7.81 ± 9.01	0.222
Protein C level (% activity), mean ± SD	41 ± 20	44 ± 19	43 ± 20	0.084
Central lab protein C class (%):				0.504
Severe deficiency	54.1	48.5	51.2	
Moderate deficiency	41.1	47.0	44.2	
Normal^†^	4.9	4.5	4.7	
Mechanical ventilation, *n *(%)	158 (76.7)	178 (78.4)	336 (77.6)	0.669
Medical history, *n *(%):				
Hypertension	93 (45.1)	118 (52.0)	211 (48.7)	0.155
Coronary artery disease	28 (13.6)	36 (15.9)	64 (14.8)	0.372
Cardiomyopathy	19 (9.2)	21 (9.3)	40 (9.2)	0.878
Diabetes mellitus	43 (20.9)	66 (29.1)	109 (25.2)	0.089
Pancreatitis	9 (4.4)	10 (4.4)	19 (4.4)	0.331
Liver disease	6 (2.9)	8 (3.5)	14 (3.2)	0.200
COPD	37 (18.0)	34 (15.0)	71 (16.4)	0.136
Malignancy	40 (19.4)	50 (22.0)	90 (20.8)	0.290
Stroke	7 (3.4)	14 (6.2)	21 (4.8)	0.139
Thrombosis	2 (1.0)	13 (5.7)	15 (3.5)	0.009
Baseline medications, *n *(%):				
Steroids for septic shock	100 (48.5)	108 (47.6)	208 (48)	0.841
Insulin	106 (51.5)	138 (60.8)	244 (56.4)	0.050
Statins	42 (20.5)	46 (20.3)	88 (20.4)	0.954
Prophylactic heparin	82 (39.8)	97 (42.7)	179 (41.3)	0.537

*Frequencies were analyzed using Pearson's chi-square test, and comparisons of continuous data were based on Type III sums of squares from ranked ANOVA models with a term for treatment.

^† ^Defined as protein C deficient based on local laboratory results.

ANOVA, analysis of variance; APACHE, acute physiology and chronic health evaluation; COPD, chronic obstructive pulmonary disease; DIC, disseminated intravascular coagulation; OD, organ dysfunction; SD, standard deviation; SOFA, sequential organ failure assessment

**Table 2:** Sites and causes of infection in the primary efficacy population

Variable	Alternative therapy (*n *= 206)	Standard therapy (*n *= 227)	Total (*n *= 433)	*P*-value*
Primary site of infection, *n *(%):				0.410
Lung	106 (51.5)	87 (38.3)	193 (44.6)	
Abdomen	46 (22.3)	64 (28.2)	110 (25.4)	
Urinary tract	26 (12.6)	28 (12.3)	54 (12.5)	
Skin	9 (4.4)	15 (6.6)	24 (5.5)	
Blood	9 (4.4)	12 (5.3)	21 (4.8)	
Other^†^	10 (4.9)	21 (9.3)	31 (7.2)	
Source of infection, *n *(%):				0.923
Community	158 (76.7)	175 (77.1)	333 (76.9)	
Nosocomial	48 (23.3)	52 (22.9)	100 (23.1)	
Type of infecting agent^‡^, *n *(%):	**(*n *= 163)**	**(*n *= 168)**	**(*n *= 331)**	
Fungal	20 (12.3)	16 (9.5)	36 (10.9)	
Gram-negative	75 (46.0)	91 (54.2)	166 (50.2)	
Gram-positive	82 (50.3)	91 (54.2)	173 (52.3)	
Mixed aerobic/anerobic	7 (4.3)	9 (5.4)	16 (4.8)	
Viral	3 (1.8)	1 (0.6)	4 (1.2)	
Other	4 (2.5)	8 (4.8)	12 (3.6)	

*Frequencies were analyzed using Pearson's chi-square test.

^†^Other sites of infection included the bone, central nervous system, head, other, pleura and reproductive tract.

^‡^All pathogens obtained from positive cultures. Patients may have had more than one infecting agent.

### Efficacy

The study met its primary objective and demonstrated that alternative therapy resulted in a greater increase in protein C level from study Day 1 to Day 7 compared with standard therapy. There was a difference in absolute change of 7% (95% confidence interval (CI) (2, 13); *P *= 0.011) (see Table 3) between the standard arm and the variable dose and duration arm. More patients randomized to alternate therapy had their final protein C increase above the lower limit of normal. This difference in protein C change persisted when we analyzed the data either (1) without imputation with the assessment restricted only to those with complete Day 1 and Day 7 data (*n *= 326), or (2) if the analysis was limited to patients where local and central protein C laboratory data matched (*n *= 302) (both predefined sensitivity analyses of the primary objective). The secondary objectives showed a similar pattern of results in both the moderate and severe deficiency subpopulations. The combined mortality for the groups demonstrated that normalization of protein C, regardless of treatment received, was associated with lower mortality (10.3%; 24/232 in patients who normalized their protein C up to Day 7 *vs *32.0%; 63/197 in patients who did not normalize; *P *< 0.0001). Furthermore, in a predefined analysis of patients where the protein C levels normalized by study Day 7 (determined by local labs), a significantly greater percentage of alternative therapy patients normalized their protein C and remained normal, and a smaller percentage did not attain a normal protein C value compared to standard therapy (60.7% *vs *51.5% and 17.0% *vs *32.2%; association *P *= 0.003), where normalization of protein C was defined as two consecutive local laboratory measurements above the lower limit of normal.

**Table 3:** Change in protein C level from study Day 1 to study Day 7 in the primary efficacy population

	Alternative therapy	Standard therapy	*P*-value*	Absolute difference in change	Two-sided 95% CI
**Primary Objective:**	***n *= 202**	***n *= 221**			
Change in PC, days 1 to 7^†^, mean activity units (%) ± SD	31 ± 29	24 ± 29	0.011	7	(2, 13)
Classification of change^‡^, *n *(%)					
No change or decreased	38 (18.8)	61 (27.6)			
Increased, but still deficient	64 (31.7)	60 (27.1)			
Increased and above LLN	100 (49.5)	100 (45.2)			
**Secondary Objective** **Moderate deficiency group:**	***n *= 171**	***n *= 175**			
Change in PC, days 1 to 7^†^, mean activity units (%) ± SD,	30 ± 29	24 ± 28	0.047	6	(0, 12)
Classification of change^‡^, *n *(%)					
No change or decreased	35 (20.5)	46 (26.3)			
Increased, but still deficient	50 (29.2)	44 (25.1)			
Increased and above LLN	86 (50.3)	85 (48.6)			
**Secondary Objective** **Severe deficiency group:**	***n *= 31**	***n *= 46**			
Change in PC, days 1 to 7^†^, mean activity units (%) ± SD,	38 ± 27	25 ± 32	0.063	13	(-1, 27)
Classification of change^‡^, *n *(%)					
No change or decreased	3 (9.7)	15 (32.6)			
Increased, but still deficient	14 (45.2)	16 (34.8)			
Increased and above LLN	14 (45.2)	15 (32.6)			

*P-value calculated by an unadjusted two-sample t-test.

^†^Change in protein C results analyzed with imputation.

^‡^Percentage of protein C change from baseline >10%. The *P*-value for protein C classification as increased in the primary objective is 0.03, calculated by a Chi-Square test.

CI, confidence interval; LLN, lower limit of normal; PC, protein C

**Figure 2:**
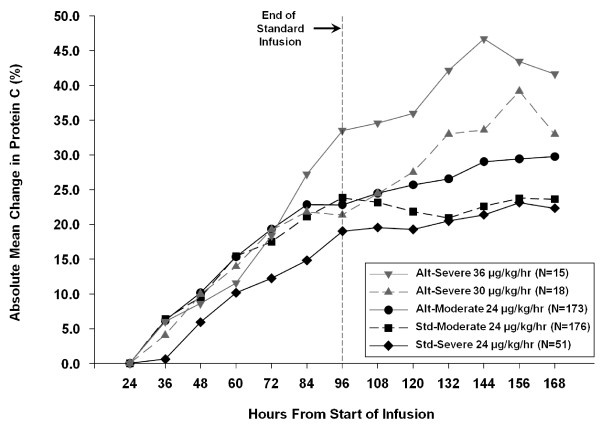
**Absolute mean change in protein C levels**. Change in mean protein C levels from study Day 1 up to study Day 7 for different therapy groups in the primary efficacy population. Alt, alternative; std, standard

Mean change in protein C levels from study Day 1 to 7 for the different therapy groups (Figure 2) demonstrated that both the higher doses and the potential for longer infusion duration increased protein C levels compared with standard therapy. Illustrating this is the fact that in the moderate strata (protein C >1/2 lower limit of normal), both treatment arms essentially received the same therapy for the first 96 hours of the study. During this time (Figure 2) changes in protein C values were similar. Only after 96 hours, when there was the potential to extend therapy in the alternate treatment arm, did the curves separate with protein C levels continuing to increase in the alternative therapy cohort.

**Figure 3:**
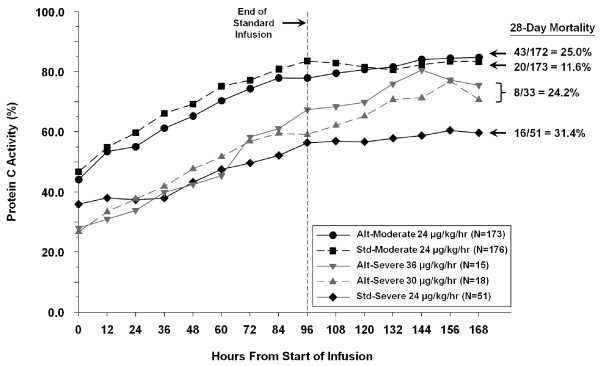
**Protein C level over time by therapy in the primary efficacy population**. Alt, alternative; std, standard.

Absolute protein C level (imputed) over time for the different therapy groups are shown in Figure 3, with associated mortality. Although the standard group starts with a higher protein C activity at baseline and at 24 hours, the alternative therapy groups show a greater increase in protein C activity.

### Mortality and safety

On the recommendation of the DMC for the study, the protocol was amended following the first interim analysis (after 209 patients were randomized) to remove the option of an infusion duration of less than 96 hours in the alternative therapy patients. Initially, alternative therapy included the option to switch to a placebo infusion if the protein C level normalized between 48 and 84 hours, and the investigator site was in agreement. Six of the patients (*n *= 22) who stopped the infusion early, had died in comparison to one patient in the standard therapy group (*n *= 33) who had continued DAA for 96 hours. The final analysis of 28-day mortality showed 6 out of 30 patients in the alternative group who had switched early to placebo had died, versus 3 out of 41 patients in the standard group who had continued DAA for 96 hours. Of note, none of the patients stratified in the severe deficiency group (protein C levels ≤1/2 the lower limit of normal) and randomized to the alternative arm switched early to placebo. At the first interim analysis, the DMC recommended that the high dose arm increase from 30 to 36 μg/kg/hr, as specified in the protocol, since there were no serious events noted in the 30 μg/kg/hr dose arm.

A difference was noted in 28-day all-cause mortality rates among the primary efficacy population between the alternative and standard therapy groups (51/205, 24.9% *vs *36/224, 16.1%; *P *= 0.03). The mortality rates stratified by therapy groups are shown in Figure 3. A low mortality rate in the moderate deficient protein C group receiving standard therapy (20/173, 11.6%) was observed. To better understand the mortality in this subgroup, we conducted a *post hoc *analysis exploring mortality by infusion duration of study drug while excluding patients who potentially switched to a placebo infusion <97 hours because of normalization of protein C levels pre-amendment. In Table 4, 28-day mortality in patients receiving an infusion of less than 97 hours (planned 96 ± 1 hour infusion) remained higher in the alternative versus standard group, despite both groups receiving the same DAA therapy. Causes of death in this patient population are also provided in Table 4.

**Table 4:** Twenty-eight-day mortality by infusion duration in the moderate protein C deficiency population

	Alternative therapy Moderate protein C deficiency 24 μg/kg/hr			Standard therapy Moderate protein C deficiency 24 μg/kg/hr		
	
Duration of study drug infusion	Number of patients	Number of deaths	Percent deaths	Number of patients	Number of deaths	Percent deaths
Total	172	43	25.0	173	20	11.6
≥97 hours*^†^	71	17 ^a^	23.9	70	8 ^b^	11.4
< 97 hours^†^	71	20 ^c^	28.2	65	9 ^d^	13.8
Patients with shorter infusions of DAA ^‡^	30	6^e^	20.0	38	3^f^	7.9

Cause of death: ^a^ Sepsis induced multiorgan failure (*n *= 5); respiratory failure (*n *= 4); refractory septic shock (*n *= 3); hemorrhage (hepatic artery) (*n *= 1); disseminated malignancy (*n *= 1); ischemic gut (*n *= 1); ischemic cardiomyopathy (*n *= 1); shock of unknown origin (*n *= 1). ^b^ Sepsis induced multi-organ failure (*n *= 5); respiratory failure (*n *= 1); refractory septic shock (*n *= 1); unknown (*n *= 1). ^c^ Sepsis induced multi-organ failure (*n *= 10); respiratory failure (*n *= 1); refractory septic shock (*n *= 8); cardial and respiratory arrest (*n *= 1). ^d^ Sepsis induced multi-organ failure (*n *= 5); respiratory failure (*n *= 2); refractory septic shock (*n *= 0); primary cardiac arrhythmia (*n *= 1); hypoxic brain injury (*n *= 1). ^e^ Sepsis induced multi-organ failure (*n *= 3); respiratory failure (*n *= 2); refractory septic shock (*n *= 1). ^f^ Sepsis induced multi-organ failure (*n *= 1); respiratory failure (*n *= 1); refractory septic shock (*n *= 1). *97 hours was used as cut off point as standard infusion time was 96 ± 1 hr. ^†^Excluding patients with shorter infusions of drotrecogin alfa (activated) (DAA). ^‡^Alternative patients potentially switched to a placebo infusion <97 hours because of normalization of protein C levels between 48 to 84 hours preamendment, while standard therapy patients received 96 hours of drotrecogin alfa (activated).

Serious bleeding events by study day in the primary efficacy population are displayed in Table 5. The majority of these events occurred during days 0 to 4 in patients stratified in the moderate deficiency group receiving alternative therapy, when these patients received the same dose and duration of DAA therapy as the standard therapy group. Three serious bleeds in the alternative therapy population occurred during days 5 to 8, when patients could potentially receive longer duration therapy. In fact, though, these bleeding events all transpired after the completion of study drug infusion. One fatal bleed in the alternative therapy group occurred at Day 24, which was not considered as study related. No serious bleeding events were observed in patients stratified in the severe deficiency group receiving higher doses and/or longer duration therapy of DAA.

**Table 5:** Serious bleeding events by study day in primary efficacy population

	Alternative therapy		Standard therapy	
	
Time period	Severe (*n *= 33) 30 to 36 μg/kg/hr	Moderate (*n *= 173) 24 μg/kg/hr	Severe (*n *= 51) 24 μg/kg/hr	Moderate (*n *= 176) 24 μg/kg/hr
Days 0 to 4	0	9 (4 GI, catheter, renal, hematoma, hemoptysis, hepatic)	0	2 (GI)
Days 5 to 8	0	3*^†^ (CNS, pleural, shock)	1 (hemoptysis)	0
Days 9 to 28	0	1^‡^ (hepatic)	0	1^†^ (CNS)
After day 28	0	1^†^ (CNS)	0	0
Total	0	14^§^	1	3

*Patients completed the study drug infusion per protocol - event occurred on the same day (*n *= 1; pleural hemorrhage) or day after (*n *= 2; cerebral hemorrhage; shock hemorrhage) infusion was completed. ^†^CNS bleeds: cerebral hemorrhage Day 7 (*n *= 1), cerebral hematoma Day 11 (*n *= 1), cerebral hemorrhage Day 32 (*n *= 1). ^‡^Fatal bleeds: arterial hemorrhage (hepatic) Day 24 following surgery, not study related (*n *= 1). ^§^One patient experienced two events on Day 2 and Day 7. CNS, central nervous system; GI, gastrointestinal

The rates of serious adverse events (including bleeding events) over the 28-day period in the primary efficacy population were 45/206 (21.8%) in alternative therapy and 27/227 (11.9%) in standard therapy (*P *= 0.007). The rates of serious thrombotic events were similar between the two groups (3/206; 1.5% in alternative *vs *2/227; 0.9% in standard; *P *= 0.672).

## Discussion

This phase 2 double-blind randomized controlled trial of a variable dose and duration of DAA demonstrates that this approach leads to higher final protein C levels. Additionally, we confirm that protein C levels correlate with survival in severe sepsis. We further demonstrate that it is possible to tailor and individualize therapy in critically ill patients with the use of bedside selected biomarkers. Finally, our findings underscore the linear pharmacodynamics of DAA and that DAA in part, although not entirely, exerts its effect through directly increasing endogenous protein C levels.

With respect to our primary endpoint, several factors merit comment. First, our conclusions regarding the connection between a variable dose and duration of DAA infusion and final protein C levels are robust. Whether analyzed with or without imputation for missing values, protein C levels remain consistently higher in patients treated under the alternative paradigm. The 7% absolute change between the two therapy groups is likely to be clinically meaningful, as in PROWESS [3] the final difference in protein C level between DAA and placebo was 7% on Day 4, and a 7.5% increase in protein C was estimated to be associated with a relative risk reduction of 15 to 20% in 28-day mortality based on logistic regression analyses. Normalization of protein C is also likely to be a clinically meaningful endpoint; a greater proportion of patients randomized to alternative therapy normalized compared to standard therapy, and as highlighted in other studies, normalization of protein C is associated with lower mortality (in RESPOND Day 28 mortality was 10.3% in patients who normalized by Day 7, compared to 32% in patients who did not normalize). Second, the raw point estimate for the effect of a tailored approach to DAA infusion is greater in the more severely protein C deficient patients (that is, 6% absolute difference in those moderately deficient *vs *13% in the severely deficient subjects). This reinforces the mechanistic connection between the alternate treatment regimen and protein C levels. Since the patients with severe protein C deficiency could potentially have the greatest increases in protein C activity given their very low starting points, one logically would predict that the relative impact of a variable dose and duration would be more extensive and thus one cannot assume that the effect of higher doses in the moderately protein C deficient group would be similar. Third, and similarly, among moderately deficient individuals protein C levels did not diverge until subjects actually could be treated differentially. Fourth, and reflecting the effect of absolute changes in protein C levels, fewer patients treated under the alternative therapy strategy had final protein C levels that either fell or failed to increase.

As noted above, the option for an extended infusion appeared to have a more modest impact than that noted with a higher dose coupled with the option for an extended duration. In part this reflects a numerical fact that there was essentially more potential for an increase in protein C values for those starting with very low protein C levels. However and perhaps more importantly, around half of subjects in the moderate deficiency group randomized to the option of an extended duration actually only required a 96 hr infusion at 24 μg/kg/hr. This observation suggests that the dose administered in PROWESS [3] and currently approved for clinical use by regulatory authorities is likely correct for most patients.

In contrast to PROWESS [3], we observed that many subjects had only moderately suppressed protein C levels after 24 hours of standard therapy. In PROWESS [3], approximately 40% of subjects had severe protein C deficiency [11] while in our study only approximately 20% had a similar deficiency. This may in part be due to the relatively smaller sample size of the current study. However, it may reflect that physicians are either identifying subjects earlier in the course of their sepsis or, perhaps, treating patients more aggressively at presentation [16]. In other respects, our population appears similar to others reported in trials either assessing novel therapies for severe sepsis or describing the epidemiology of this syndrome. For example, the vast majority of subjects we enrolled required both vasopressors and mechanical ventilation and the lung was the most common site for infection.

With respect to safety, the overall rates of serious bleeding events mirror those seen in previous DAA studies (PROWESS [3], ENHANCE [17]). However, in the moderately protein C deficiency group, there were higher rates of serious bleeding in patients receiving alternative therapy, which is difficult to explain as the majority of these events occur during the first four days when patients are receiving the same treatment. This is most likely a chance finding related to small sample size, as there appears to be no clear reason why the bleeding rates would be different over a time when both randomized groups were receiving the same therapy. It is reassuring that no serious bleeding events were related to higher doses; however, the numbers of patients receiving higher doses were relatively small and ultimately a larger study would be required to better quantify how bleeding relates to a higher dose and/or longer duration of DAA.

**Figure 4:**
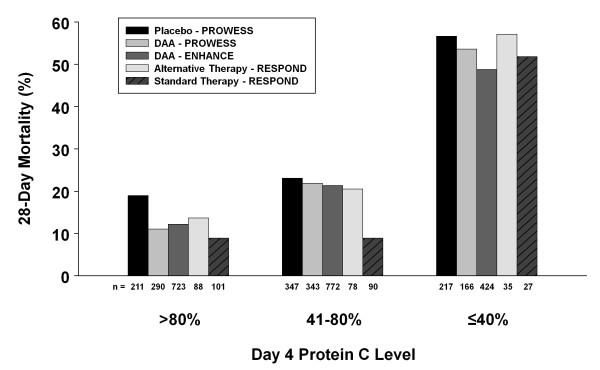
**Comparison between studies of 28-day mortality by Day 4 protein C level**. Twenty-eight-day mortality is shown based on Day 4 protein C levels by categories: normal (> 80%); moderately deficient (41 to 80%); and severely deficient (≤40%) for PROWESS [3], ENHANCE [17] (both reported by Vangerow *et al*. 2007 [14]), and RESPOND. The number (n) under each column is the total number of patients in each category. DAA, drotrecogin alfa (activated).

As with the serious bleeding events, the overall mortality was higher in alternative therapy patients with moderate protein C deficiency. Upon distillation of the therapy groups, it can be seen that the 28-day mortality rates were similar to those seen in the DAA-treated groups from PROWESS [3] and ENHANCE [17] (24.7% and 25.3% respectively) except for patients stratified as moderately deficient in the standard paradigm, as depicted in Figure 4. The reason for this unseemingly low mortality rate within an obviously sick group of patients is unclear. What is interesting is that in the moderately deficient groups who all received the same dose of DAA, whether patients received a shorter infusion duration, the standard infusion duration, or a longer duration of DAA (as highlighted in Table 4, 28-day mortality by infusion duration), the mortality was higher in the alternative group compared to standard, which would imply that these differing mortality rates are not due to the intervention of DAA itself. It is also of note that higher mortality with alternative therapy was not seen in the severe deficiency strata, where the alternative therapy received the longest infusion durations and highest overall exposure. Most patients died of sepsis related causes, and there were no deaths thought to be related to study drug. However, it must be remembered this is a phase 2 trial not powered for mortality and the small sample size in the alternative therapy groups renders these mortality results unreliable. Nonetheless it was disappointing that no overall trend for a mortality improvement was seen with alternative therapy. Shorter infusions of DAA have been proposed in patients based on clinical markers [18,19]. Based on the experience of this trial, we would not recommend shorter infusions of DAA.

One key unique aspect of our project was the tailoring of treatment based on serial biomarker measurements. Very few studies, in either hospitalized patients or critically ill subjects, have attempted to individualize a therapeutic intervention based on both initial values and their sequential evaluation over time. Although reliance on procalcitonin to guide antibiotic therapy duration represents employment of a biomarker [20], this assay simply helps the clinician determine when to discontinue antibiotics. Neither the dose of antibiotic nor the class of antibiotic is affected by a procalcitonin level. Our protocol resulted in frequent and direct adjustments in DAA infusions and affords a potentially novel way to shift paradigms in how we treat critically ill patients. Thus, as a proof of concept, our trial emphasizes that it is indeed possible to titrate and individualize novel therapies in critically ill patients. Furthermore, it is possible to study such interventions in a rigorous fashion. Currently, however, we would not recommend titration of DAA outside the clinical trial setting.

It is worth noting that the restoration of normal protein C measurements does not necessarily account for all of the treatment effect of DAA. In an analysis from PROWESS [3] and ENHANCE [17] patients to test which biomarkers could serve as surrogate end-points by predicting clinical benefit, restoration to normal protein C level accounted for 57% of the treatment effect [9]. Indeed, the key test of protein C as a clinically relevant biomarker with which to titrate DAA therapy will come from a future phase 3 study powered to investigate if normalization of plasma protein C levels by DAA correlates with patient benefit.

Our study has some limitations. These include stratification of patients to moderate and severe deficiency at 24 hours rather than at baseline, which delayed some patients receiving higher doses, and also resulted in an imbalance of numbers between the severe deficiency alternative and standard therapy groups. This study design was incorporated to ensure that only patients who remained at high-risk of early death despite 24 hours of standard therapy were exposed to higher doses. However, now that this study has collected additional safety information related to higher doses this would most likely not be repeated in potential future studies. During this 24-hour common treatment period, the slight imbalance in protein C deficiency noted at baseline between treatment group became statistically significant in the moderately deficient subgroups. Alternative therapy only differed from standard therapy after study Day 4, so there was no opportunity for alternative therapy to improve protein C levels until after this point in time and although the percent change was higher for alternative therapy compared to standard, absolute levels actually remained lower than standard therapy for the majority of the infusion period (Figure 3). Given the link demonstrated in this and other studies between lower protein C levels and higher mortality, it is possible that these baseline differences in protein C that remained for much of the infusion period, may have contributed, at least in part, to the observed mortality differences. Assumptions during study design regarding the percentage of patients who would be stratified as severely and moderately protein C deficient, and the percentage of patients who would require a longer infusion duration to normalize their protein C, proved to be incorrect. This led to a smaller than expected proportion of patients receiving alternative therapy that differed from standard therapy, adding to the complexity of interpreting the safety results. There were also a relatively small number of patients exposed to higher doses because fewer patients were classified with severe protein C deficiency at 24 hours than predicted from the PROWESS data [14], which limits the conclusions that can be drawn from this subgroup. The reliance on local protein C assays to stratify patients led to some misclassification of protein C values between local and central laboratories and the potential relevance of this warrants further investigation. Although mortality differences were noted, the study was not primarily designed or powered to detect 28-day mortality differences between subgroups. Our study also has some notable strengths. First, the primary objective was based on a single central laboratory protein C assay; however, the results were similar whether based on central or local laboratory data. Also predefined sensitivity analyses confirmed our primary efficacy result.

## Conclusions

This phase 2 trial met its primary objective of improved protein C levels in patients receiving alternative therapy and is part of an evolving picture which strives to explore the concept of tailored therapy using a biomarker in sepsis. It has confirmed that protein C levels are linked to outcomes and has explored the paradigm that would allow more patients to have increased protein C levels. Finally, it has also provided valuable information to be incorporated into potential future trials which could further characterize the potential clinical benefit and risk associated with higher doses and/or longer infusions of DAA.

## Key messages

• Since change in protein C levels over time are highly correlated with outcomes, this phase 2 trial was designed to explore use of protein C levels as a potential biomarker in severe sepsis to optimize drotrecogin alfa (activated) therapy for individual patients.

• The RESPOND study met its primary objective, demonstrating that patients with multiple organ dysfunction and protein C deficiency have greater improvements in protein C with alternative therapy (higher dose and/or variable duration) compared to standard drotrecogin alfa (activated) therapy.

• This study confirms, as seen in other studies, that protein C normalization correlates with survival in severe sepsis.

• It may be possible to tailor drotrecogin alfa (activated) therapy in critically ill patients with the use of a real-time biomarker.

• RESPOND provides valuable information to help decide the most appropriate aspects of "alternative therapy" to incorporate into possible future studies, aimed at tailoring drotrecogin alfa (activated) therapy to individual patient requirements based on protein C levels; until such additional studies are performed, titration of drotrecogin alfa (activated) outside the clinical trial setting is not recommended.

## Abbreviations

Alt: alternative; ANOVA: analysis of variance; APACHE: acute physiology and chronic health evaluation; CI: confidence interval; CNS: central nervous system; COPD: chronic obstructive pulmonary disease; DAA: drotrecogin alfa (activated); DIC: disseminated intravascular coagulation; DMC: data monitoring committee; ENHANCE: Extended evaluation of recombinant human activated protein C; GI: gastrointestinal; IVRS: interactive voice response system; LLN: lower limit of normal; OD: organ dysfunction: PC: protein C; PROWESS: Recombinant human activated PROtein C Worldwide Evaluation in Severe Sepsis; SD: standard deviation; SOFA: sequential organ failure assessment; Std: standard.

## Competing interests

Drs. Shorr, Artigas, Tenhunen, Wyncoll, Mercier, Francois, Vincent and Reinhart have participated as investigators in Eli Lilly and Company sponsored trials. Dr. Wyncoll has served as a consultant to and given paid talks for Eli Lilly and Company. Drs. Janes, Vangerow, Heiselman, Leishman and Ms. Zhu are employees and stockholders of Eli Lilly and Company.

## Authors' contributions

All authors (apart from AL) participated in the conception, design or conduct of the study. All authors participated in the analysis and interpretation of data, with YZ performing the statistical analysis, and all authors helped draft, critically revised, and read and approved the final manuscript.

## Supplementary Material

Additional file 1 **Supplementary data**. A word document containing the following tables and figure: Table S1: Disease diagnostic criteria; Table S2: Summary of exclusion criteria; Table S3: Definitions of protein C deficiency; Table S4: Expected versus actual study parameters; Figure S1: Simplified RESPOND study design. Click here for file
