# Supplementary material for: Randomized trial evaluating serial protein C levels in severe sepsis patients treated with variable doses of drotrecogin alfa (activated)
Source: Crit Care. 2010 Dec 21;14(6):R229. doi: 10.1186/cc9382 (PMC3219981; doi:10.1186/cc9382)
Supplement: Additional file 1 — Supplementary data. A word document containing the following tables and figure: Table S1: Disease diagnostic criteria; Table S2: Summary of exclusion criteria; Table S3: Definitions of protein C deficiency; Table S4: Expected versus actual study parameters; Figure S1: Simplified RESPOND study design. [file cc9382-S1.DOC]

**ADDITIONAL FILES**

**Randomized trial evaluating serial protein C levels in severe sepsis patients treated with variable doses of drotrecogin alfa (activated) additional file 1.**

**Table S1.** Disease Diagnostic Criteria.

| **PRESENCE OF A SUSPECTED OR PROVEN INFECTION:**  Patients with a suspected infection must have evidence of an infection, such as white blood cells in a normally sterile body fluid, perforated viscus, chest x-ray consistent with pneumonia and associated with purulent sputum production, or a clinical syndrome associated with a high probability of infection, for example, purpura fulminans or ascending cholangitis.  **ORGAN DYSUNCTION DEFINITION:**  **Cardiovascular:** An arterial systolic blood pressure (SBP) of ≤90 mm Hg or a mean arterial pressure (MAP) ≤70 mm Hg for at least 1 hour despite adequate fluid resuscitation, adequate intravascular volume status, or the need for vasopressors to maintain SBP ≥90 mm Hg or MAP ≥70 mm Hg.  Adequate fluid resuscitation or adequate intravascular volume is defined as one or more of the following: (a) the administration of an intravenous fluid bolus (≥500 mL of crystalloid solution, ≥20 g of albumin, or ≥200 mL of other colloid administered over 30 minutes or less); (b) pulmonary arterial wedge pressure ≥12 mm Hg; or (c) central venous pressure ≥8 mm Hg.  Vasopressors are defined as the following: (a) dopamine ≥5 µg/kg/min or (b) norepinephrine, epinephrine, phenylephrine, or vasopressin at any dose. Dobutamine or dopexamine are not considered vasopressors.  **Renal:** Average output <0.5 mL/kg/h for 1 hour despite adequate fluid resuscitation (defined above).  In the presence of preexisting impairment of renal function (defined as a serum creatine concentration >2 times the upper limit of the normal reference range for the institution prior to the onset of sepsis), the patient must meet two of the other four organ dysfunction criteria.  **Respiratory:** Evidence of acute pulmonary dysfunction: PaO2/FiO2 ≤250 (adjusted for altitude) and, if measured, a pulmonary capillary wedge pressure not suggestive of central volume overload. If the lung is also the suspected site of infection, the patient must have a PaO2/FiO2 <200.  PaO2 altitude adjustment: Sites at altitudes above 1000 feet should multiply the observed PaO2 by (760/LBP) - where LBP is the local barometric pressure - before calculating the PaO2/FiO2 ratio.  **Hematology:** Platelet count <80,000/mm^3^ or a 50% decrease in platelet count from the highest value recorded over the 3 days prior to study entry.  **Unexplained metabolic acidosis:** Defined by (1) pH ≤7.30 or base deficit ≥5.0 mEq/L and (2) a plasma lactate level >1.5 times the upper limit of normal for the reporting laboratory.  Measurement of pH or base deficit and lactate level should occur within a clinically relevant time interval such that a casual relationship exists between the measured values. |
| --- |

**Table S2.** Summary of Exclusion Criteria

| - Documented multiple organ dysfunction for greater than 24 hours prior to the start of study drug or the first documented sepsis-induced organ dysfunction occurred greater than 36 hours prior to the start of study drug - Weight <30 kg or >135 kg - Platelet count <30,000/mm^3^ - Active internal bleeding or increased risk for bleeding, for example:   (a) any major surgery, defined as surgery that requires general or spinal anesthesia, performed within the 12-hour period immediately preceding the drotrecogin alfa (activated) infusion, or any postoperative patient who demonstrates evidence of active bleeding, or any patient with planned or anticipated surgery during the infusion period (for example, patients with staged surgeries or burn patients with planned excisions and grafting during the infusion period) (Note: peritoneal lavage alone is not considered planned surgery)  (b) biopsy or surgical procedure of a closed-space within the 12 hours immediately preceding the drotrecogin alfa (activated) infusion where there is a high risk of significant bleeding and where it would not be possible to control bleeding by external pressure  (c) history (within the previous 3 months) of stroke or severe head trauma that required hospitalization or intracranial surgery  (d) history of intracranial arteriovenous malformation, cerebral aneurysm, or central nervous system mass lesion  (e) patients with an epidural catheter or who are anticipated to receive an epidural catheter during drotrecogin alfa (activated) infusion  (f) history of congenital bleeding diatheses (for example, hemophilia)  (g) gastrointestinal bleeding within the 6 weeks prior to study entry that required medical intervention unless definitive endoscopic procedure or surgery has been performed  (h) trauma patients at increased risk of bleeding (for example, flail chest; significant contusion to lung, liver, or spleen; retroperitoneal bleed; pelvic fracture; compartment syndrome)  (i) patients with known esophageal varices, chronic jaundice, cirrhosis, or chronic ascites   - Concurrent need for any of the following medications during the drotrecogin alfa (activated) infusion:   (a) therapeutic heparin, defined as unfractionated heparin >15,000 units/day within 8 hours of study entry or low molecular weight heparin used at any dose higher or more frequent than the recommended dose in the product label for prophylaxis within 12 hours of study entry  (b) warfarin, if used within 7 days of study entry or warfarin-type medications within <5 half-lives at the time of study entry and where the prothrombin time (PT) is prolonged beyond the upper limit of normal for the institution  (c) antiplatelets such as ticlopidine, clopidogrel, or acetylsalicylic acid (ASA) >650 mg/day or compounds that contain ASA >650 mg/day within 3 days prior to study entry  (d) thrombolytic therapy (unless used to treat an intra-catheter thrombosis; however, care should be taken to avoid systemic administration) if used within 3 days of study enrollment (for example, streptokinase, tPA, rPA, and urokinase)  (e) glycoprotein IIb/IIIa receptor antagonists within 7 days of study entry  (f) antithrombin infusion of >10,000 units within 12 hours of study entry  (g) protein C concentrate infusion within 24 hours of study entry  (h) other anticoagulants, such as direct thrombin inhibitors (for example, hirudin, argatroban, bivalirudin, desirudin, lepirudin, ximelegatran, or melegatran) and factor Xa inhibitors (for example, fondaparinux) and other synthetic heparinoids within <5 half-lives of study entry  (i) recombinant factor VIIa within the past 30 days   - Patient not expected to survive 28 days given their preexisting uncorrectable medical condition - HIV/AIDS patients with known end-stage processes (for example, progressive multi-focal leukoencephalopathy [PML], mycobacterium avium complex [MAC], Epstein-Barr virus [EBV], or lymphoma, or a known CD4 count <50 cells/mm^3^) - Patient is moribund and death is perceived to be imminent (within 24 hours) - Patient’s family or primary physician not committed to aggressive management of the patient, or an advanced directive to withhold life-sustaining treatment, with the exception of cardiopulmonary resuscitation - Received treatment within the last 30 days with drotrecogin alfa (activated) - Pregnancy or breast-feeding |
| --- |

**Table S3.** Definitions of Protein C Deficiency

|  | **Type of Test** | **PC deficiency**  **(< LLN)** | **Severe PC deficiency**  **(≤ ½ LLN)** | **Moderate PC deficiency**  **(> ½ LLN)** |
| --- | --- | --- | --- | --- |
| Central Lab* | Staclot | ≤80% activity | ≤40% activity | >40% activity |
| Local Lab^†^ | Biosite | ≤2.55 µg/mL | ≤1.275 µg/mL | >1.275 µg/mL |
| Local Lab^†^ | Stachrom | 70%-90% activity | 35%-45% | 46%-89% |

*Central lab results used for primary analysis; not available to investigators; not used for treatment stratification

^†^Local lab sites used for treatment stratification; made decisions related to completion of study drug infusion

PC=protein C, LLN=lower limit of normal

**Table S4.** Expected versus Actual Study Parameters

|  | **Expected (% of patients)** | **Actual (% of patients)** |
| --- | --- | --- |
| Randomized patients who receive study drug during the common therapy lead-in period, but did not continue to randomized therapy due to death or a serious adverse event | 6% | 11% |
| Patients receiving randomized therapy (primary efficacy-population patients) stratified as: |  |  |
| Moderate protein C deficiency | 60% | 80% |
| Severe protein C deficiency | 40% | 20% |
| Alternative therapy patients in the moderate protein C deficiency strata who received an infusion longer than 97 hours | 70% to 75% | 46% |

PC=protein C, LLN=lower limit of normal, DAA=drotrecogin alfa (activated)

**Figure S1.** Simplified RESPOND Study Design
